# Institutional Notes and News

**Published:** 1910-04-09

**Authors:** 


### Institutional Notes and News.

**A windfall for Staffordshire Infirmary.**

The Gainsborough portrait of Mr. John Eld, the first treasurer of the Staffordshire General Infirmary, is to be sold. The story of its hanging for 140 years unsuspected of genius in the board room of the hospital is now, of course, well known. The Earl of Lichfield, who presided at the annual meeting of subscribers, pointed out how difficult it would be to keep a picture of recognised value, and how greatly the infirmary would benefit by the substantial sum it was likely to realise. A good copy, he hoped, and the fine old original frame would be retained in the board room. The hospital's expenditure last year was £3,654, and its income £3,168, leaving a balance on the wrong side of £485. This unfortunately is not the first of these adverse balances, which form to-day a grand total of £1,079. As last year moreover the infirmary received no legacies the discovery of an unsuspected treasure in the board room has come at a very fortunate time.

**Birmingham and Midland Women's Hospital.**

It is not often that the success of a hospital's treatment is made a matter for criticism, but an important place at the annual meeting of the Birmingham and Midland Hospital for Women the other day was given to the consideration of a criticism of this kind. Last year the hospital's death rate of the most difficult cases was 2.5, and this, it would seem, has led local opinion to imagine that the acting surgeons "picked their cases," and tended to prefer their professional reputation to the patient's welfare. Mr. G. Hookham referred to the matter in these words: "The charge has its flattering side, as it means that people outside the hospital cannot account for our results in any other way." Mr. Hookham then took the straight course of inviting any who had any evidence in support of the popular charge to communicate with him under guarantee of secrecy, so that the evidence could be dealt impartially with. No honourable man, Mr. Hookham pointed out, could refuse this challenge and then continue to repeat the horrible charge. We are reminded of Beau Nash's rule, still to be read in the Pump Room at Bath, that all repeaters of scandal be taken for the authors of it. The in-patient department last year received 834 patients, and there were 3,867 new out-patients. During the five years from 1904 to 1908, 1,939 operations, involving abdominal section, have been performed; in 42 cases the patients died, the death-rate therefore was only 2.2 per cent. It should be added that the new ward designed for serious cases, which was opened on the first day of the New Year, has already proved its usefulness.

**New Dental Hospital, Liverpool.**

The last fourteen months at Liverpool have been energetically occupied in completing the new dental hospital, which has cost £10,000 to build. Lord Derby opened the building and presided afterwards at the meeting. Apart from architectural considerations, there is a "feature" of the new dental hospital which is worthy of notice, and that is the attempt it is making to discriminate between the economic classes of its patients. The right to pay is being recognised and patients will have the opportunity of contributing according to their means. Of the £10,000 already referred to, all but £1,500 has been subscribed, and it is noteworthy that the dental staff was responsible for £1,000 towards the hospital's equipment. Although the dental hospital is new in every radical sense of the word, it is the living and fresh survival of an older institution, which was founded in 1860, and therefore it may be said to complete its jubilee and start its career at the same time!

**Hornsey Cottage Hospital.**

The first annual meeting of the new Hornsey Cottage Hospital had the satisfaction of recording that a legacy of £1,000 had been received during the year for the purpose of endowing a bed to be called, after the donor, the "Slater" bed. The way in which the number of occupied beds had increased every week since the opening showed that the unfortunate events which had delayed that ceremony had not been overcome a moment too soon. Twelve patients were in the wards at the time of the meeting, and it is hoped that annual subscriptions will realise about £450. Only £500 is required to complete the purchase of the freehold.

**Children's Hospital, Pendlebury.**

The courageous policy of offering to women, on the same conditions as to men, the post of assistant medical officer at the out-patients' department of the Manchester Children's Hospital, Pendlebury, was tried a year ago, and is referred to in the recent annual report. Mr. George H. Leigh, who presided, remarked that a children's hospital gave the best opportunity for an experiment of the kind. Time would show whether or not the principle should be extended. In suitable cases the parents of the children are asked to contribute to the hospital's funds, and there is the additional justification for this in the fact that the hospital's wards last year were fully occupied. The medical report, indeed, stated that the hospital's usefulness could be extended if further funds could be secured.

**North Ormesby Hospital.**

If the figures in the annual report of the North Ormesby Hospital are analysed, there seems to be some light thrown upon the unfortunate financial position of the hospital which is there disclosed. The daily average number of occupied beds last year was 73, and the number of in-patients was 1091. Of these, it should be noticed, 579 came from Middlesbrough, leaving roughly a balance of one-third only from the place from which the institution takes its name. The policy, therefore, which these columns have more than once recorded in dealing with annual reports all over the country, of discriminating not only between the incomes of patients, but between the areas from which they come, should be tried in this case. Middlesbrough should be made to recognise that its obligations are three times as heavy as those of North Ormesby. It is a fact of this kind that affords a starting-point for efficient hospital advertising. Were this done the total deficiency on the ordinary and extraordinary expenditure would not remain at the £1,000 which roughly expresses them now, particularly as the annual subscriptions increased last year by £73. This means that there is a good charitable soil to be worked upon. The figures of the accounts were for total income £5,154, ordinary expenditure £5,722, and extraordinary expenditure £352. It should be added that last year the hospital was painted—an expense from which there is no escape. The "Elizabeth Caroline Burn" wing was finished, and there, too, the question of maintenance expenses immediately recurred. Lately a patients' library has been started by Mr. J. W. Pennyman. The new *x*-ray apparatus is a present from Mr. Alfred O. Cochrane, the treasurer, whose generosity in other ways, particularly to the building fund, has been continual.

